# A concurrent bioceramic colloid design for dental crowns additive manufacturing by vat photopolymerization

**DOI:** 10.1038/s41598-025-24100-w

**Published:** 2025-11-07

**Authors:** Adel Osama, Noha Fouda, Mohamed T. Eraky

**Affiliations:** 1 Production Engineering and Mechanical Design Department, Faculty of Engineering, Mansoura University, P.O. 35516, Mansoura, Egypt; 2 Faculty of Engineering, Mansoura National University, Mansoura, Egypt

**Keywords:** Dental crowns, Stabilized zirconia, Materials design, Response surface methodology, Engineering, Materials science

## Abstract

The subtractive manufacturing of bioceramic dental crowns is standard in the dental sector. Still, it is accompanied by many demerits such as raw material waste, tool wear, high cost, and difficulty in handling complex geometry. Researchers direct their efforts toward additive manufacturing due to its capability to produce products with complex geometry and low material waste. This research aims to develop a concurrent engineering approach based on waste minimization of bioceramic powder extracted from subtractive manufacturing and reuse it as a raw material mixed with a polymer resin and a dispersing agent under an indirect mixing strategy to form a hybrid ceramic colloid for bioceramic additive manufacturing. Our approach consists of six stages: raw powder characterization in which particle size, material compositions, crystalline structure, and unit cell dimensions are determined; ceramic colloid design in which ceramic colloid performance based on layer thickness, ceramic powder vol% is optimized using fuzzy-entropy and fuzzy-topsis methods; indirect mixing strategy; 3d printing of the sample; green part characterization, and manufacturing cost estimation. The characterized powder has an average particle size of 91 nm, the majority of its crystalline structure is in the tetragonal phase with unit cell dimensions in angstrom of 'a' that is equal to 3.59 and ‘c’ that is equal to 5.16, the optimum bioceramic colloid is achieved at 1% wt of ceramic powder and a layer thickness of 25 microns with an average stability of 30.2 mv. The ceramic colloid is fed into a 3d printer, and the ceramic part is additively manufactured with good homogeneity and much lower cost than subtractive manufactured dental crowns. The ceramic-colloid stability, homogeneity, and layer thickness control are good indicators of the success or failure of the additively manufactured parts.

## Introduction

Teeth damage has strong implications on health. So the replacement of the damaged tissues using artificial materials, capable of withstanding the severe mechanical, chemical, thermal, and oral requirements is a critical issue that researchers in the dental sector direct their effort towards it. Dental ceramic is defined according to ISO 6872 as an inorganic, non-metallic material that specifically formulated to form the whole or part of a dental restoration or prosthesis according to the manufacturers’ instructions^1^. Dental ceramic materials are a subgroup of biomaterials that are classified into two major types, the first type is the low-toughness ceramics such as leucite ceramic that have low flexural strength, brittle behaviour, low fracture toughness and low flexural strength.so they are limited to anterior restorations, the second type is high toughness ceramics such as aiumina and zirconia that have optimal mechanical, aesthetic, physical and biocompatible behavior that makes it suitable for dental purposes such as root repair materials^2,3^. Zirconia-based materials are generically utilized for dental fields and dental implants, especially partially stabilized zirconia with yttria stabilizer that is manufactured from fine particles of Zirconia (ZrO2) with 3–5 vol % Yttria (Y2O3) stabilizer content, utilized and characterized as dental bioceramic materials, more yttria is added to improve aesthetics and stabilize bioceramic material but increasing yttria content adversely affects the strength and fracture toughness of bioceramic material. Hence, stabilizers content control becomes a decision-making problem^4,5^. Ceramics are materials with high brittleness, so conventional manufacturing technologies are limited and not suitable for shaping brittle materials when compared to other material types, such as metals and polymers. Moreover, conventional manufacturing technologies involve long processing times that decrease productivity. Ceramic elements with complex features couldn’t be produced through conventional technologies. However, CNC milling is common in manufacturing ceramic dental crowns; it has a lot of demerits, such as high material waste, high cost, tool wear, and difficulty handling with highly complex geometry^6^. So researchers direct their effort to a new technology called additive manufacturing, which produces three-dimensional parts based on a layer-by-layer approach to overcome all the subtractive manufacturing drawbacks^7^. The American Society for Testing and Materials classified additive manufacturing technologies into various processes used to form ceramic components^8^. Vat photopolymerization (VPP) is one of the most common technologies of additive manufacturing based on single material fabrication, it demonstrates superiority in a wide range of applications, and it was developed based on a chemical reaction called photopolymerization, the standard material used with this process are polymers but researchers in recent years used this process for multi material fabrication based on concept of Nanocomposites by developing an approach that is subdivided into three stages: preprocessing stage that includes design of the hybrid colloids or slurry that is consists of liquid polymers as matrix and Nano ceramic powder as reinforcement; processing stage that includes optimizing process performance parameters, and characterization stage that includes measuring homogeneity, flexural strength and surface roughness of hybrid ceramic parts that is based on VPP multi material fabrication comparing it with polymeric parts that is based on VPP single material fabrication^9,10^.

The major step in ceramic materials additively manufactured by VPP is the preprocessing stage which included the ceramic colloids preparation and design that achieve the requirements of the process. These colloids or suspensions are favorable in case of achieving three targets that are high ceramic loading (to ensure low porosity and high flexural strength), high stability, and low viscosity (as higher viscosities prevent the recoating of homogenous layers)^11^. The concept of concurrent engineering as a way for improving the performance of preprocessing bioceramic additive manufacturing could be utilized to achieve best quality, minimize waste, minimize cost, and reduce manufacturing time^12^. Stabilized zirconia powder in nanoscale that is extracted from CNC milling as a waste could be utilized as a filler to form a developed resin that could be used in ceramic additive manufacturing^13^.

In this regard, several scientific works in studying the performance parameters that control the performance of a ceramic colloid are analyzed below, in addition to some other works in bioceramics additive manufacturing.

Italo et al.^11^ prepared a homogenous ceramic slurry with high solid loading and low viscosity, they noticed that a solid loading of at least 40vol% is required to avoid post-processing defects and avoid high shrinkage, they also indicated that the higher the solid loading the higher the viscosity which affects badly on quality of additively manufactured part, they also noticed that Disperbyk111 is a good dispersant for formulation of stable additively manufactured ceramic parts.

Ning et al.^14^ studied the effect of solid loading, particle size, dispersant content, and gelling agent content on the stability of three-dimensional network structure, and noticed that the stability of the slurry decreased with large particle size especially when the loss factor was greater than one, and slurry structure was strengthened at small particle size, and flocculation tendency was improved; they also noticed that increasing solid loading improved the stability of the slurry but also increased the flocculation phenomenon, they also noticed that increasing dispersant content improve the stability of the slurry, and when the dispersant is more than optimal content, the redundant dispersant molecules were freely dispersed in the resin forming bridges between particles and that improve the stability of the slurry but on the other hand increases the viscosity of the slurry.

Insup et al.^15^ studied the effect of dispersant concentration (BYK142) on the quality and precision of additively manufactured parts, and they noticed that the ceramic suspension containing optimal dispersant concentration (2 wt%) showed the lowest viscosity with the strongest shear thining behavior, the slowest sedimentation rate, and the highest dispersion stability.

Mingxuan et al.^16^ prepared a high solid loading slurry for ceramic additive manufacturing, and noticed that with solid loading (45 vol %), achieved considerable stability with viscosity less than 3pa.sec and minimum sedimentation over seven days. Keqiang et al.^17^ provided a new process for additive manufacturing ceramic parts (pure ZrO_2_) with high performance (high solid loading with 55 vol% and low viscosity below 3pa.sec) but cracks formed during sintering process. Bartolomeo et al.^18^ indicated the main parameters that affect the mechanical properties of additively manufactured ceramic parts and noticed that increasing the solid loading decreased the linear shrinkage and improved the flexural strength but viscosity increased which adversely affects printing quality.

Jeewhan et al.^19^ discussed how dispersants affect additively manufactured ceramic parts, and noticed that both BYK111, BYK180 showed better performances than others because of their lower volatilities under normal temperature conditions during printing process, they also indicated that dispersant with optimum percentage (BYK111(3 wt%), BYK180(4 wt%)) could break down agglomerated particles and improve the flow ability by steric stabilization. However, an excessive concentration of the dispersant in the ceramic slurry could cause flocculation that leads to slurry’s fluidity deterioration.

**Fig. 1:**
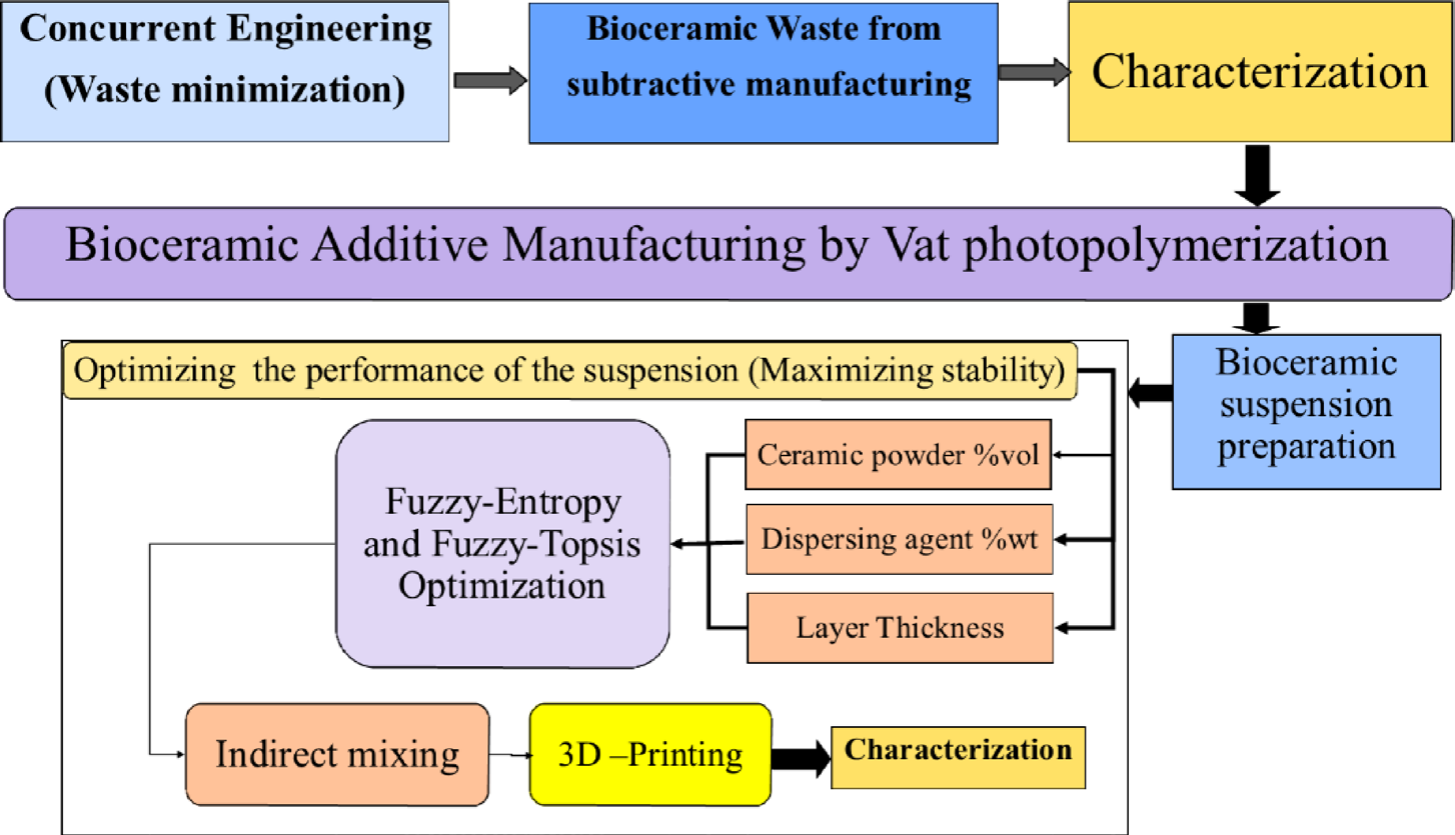
The scheme of the temporary restorations proposed model.

Although each of these previous studies has provided valuable information, covering the design of bioceramic for manufacturing but they suffer from several problems such as low printed quality, high cost, and long heat treatment time, and these problems could be treated by utilizing the concept of concurrent engineering that is applied by following steps that are firstly, utilizing the ceramic powder waste resulted from subtractive manufacturing as raw material for additive manufacturing as a method for cost minimization. Secondly, achieving high-performance nanoscale ceramic colloid instead of micro-scale. The current study aims to design a model that is schematically shown in Fig. 1 with the target of achieving high-performance nanoscale ceramic colloid based on a concurrent engineering approach for additive manufacturing of high-quality bioceramic parts, the concurrent design approach is a function of the following steps: Firstly, Raw material is characterized using XRD, TEM, SEM, and EDX, then a variety of colloids prepared and filtered using Fuzzy-Entropy and Fuzzy-Topsis to optimize viscosity and stability, Secondly, the optimized colloid has been submitted to an indirect mixing strategy then, 3d printing of the optimized colloid. Finally, green part characterization is for evaluating the homogeneity of the manufactured part. Overall, the proposed model will help dental manufacturers optimize the design and selection of dental crown material.

## Materials and methods

### Raw materials characterization

We start our study with the characterization of a stabilized zirconia powder with 4 mol% yttria which is the waste of zirconia blocks used in subtractive manufacturing, the characterization is subdivided into stages, the first stage is a morphological structure and particle size determination that is performed via high-resolution transmission electron microscope (HRTEM, JEOL JEM-2100 at an accelerating voltage of 200 kV) and Field emission Scanning electron microscope (FESEM, Quattro S, Thermo Scientific) shown in Figs. 8, 9, data in Figs. 8, 9 are extracted and statistically analyzed using Imagej and origin pro soft wares, and results shown in Table 1, Figs. 10, and 11indicated that the average particle size is nearly 91 nm and data is normally distributed. Crystalline structure is an indication of solid-state material properties^20^ so the second stage in our characterization plan is the crystalline structure and phase identification of the synthesized materials that are shown in Figs. 12, 13, Table 1 and were investigated using Empyrean Malverpanalytical, Netherland X-ray diffraction (XRD), with 2-Theta (5.0°—85°), with step size 2-Theta: 0.04 and at (Kα) = 1.54060°. Analysis of XRD data shown in Table indicates that the majority presence of tetragonal zirconia crystalline structure and the presence of the crystalline tetragonal zirconia is certified by diffraction peaks with numbers of (3,5,6,8,11,13,15,16,17,18,19) and that major existence of tetragonal phase in sample is due to amount of yttria stabilizer(4 mol%) doped in zirconium dioxide. The zirconia monoclinic structure that has low toughness and hardness^21^ was found in prepared samples at the diffraction peaks with numbers of (1,2,4,7,9,10,12,14), and that doesn’t constitute a problem as this sample will be used in making a ceramic colloid for additive manufacturing forming a green body and that green body could be used in temporarily restorations in case of low ceramic loading that doesn’t require excellent mechanical properties such as in case of permanent restorations, and also could be used in permanent restorations in case of high ceramic loading in which samples will be sintered to eliminate presence of monoclinic phase and improve mechanical and aesthetic properties of the additively manufactured sample.

**Fig. 2:**
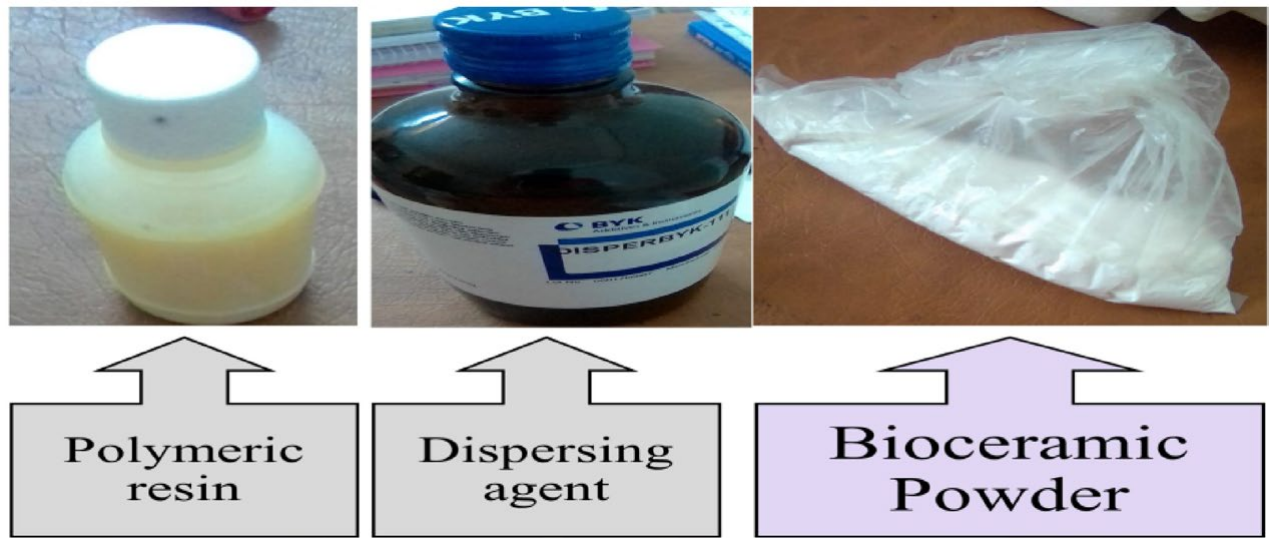
The raw materials of the ceramic colloid.

**Table 1:** The key performance indicators of the four types of the ceramic colloids at particle size diameter 90 nm, ceramic powder refractive index 2.1, resin refractive index 1.2 and wavelength 405 nm^31,32^.

Key performance indicator	Colloid 1	Colloid2	Colloid3	Colloid 4
Weight percentage of the ceramic powder %	20	10	5	1
Volume percentage of the ceramic powder %	3.333	1.667	0.833	0.167
Ideal interparticle distance (h)(nm)(Eq. 4)	135	194	268	522
Griffith factor Q	0.270	0.388	0.536	1.044
Ideal Depth of penetration(Dp)(micron)	6.5	9	13	34
Actual depth of penetration(0.75 Ideal depth of penetration)	5	4	10	25

**Fig. 3:**
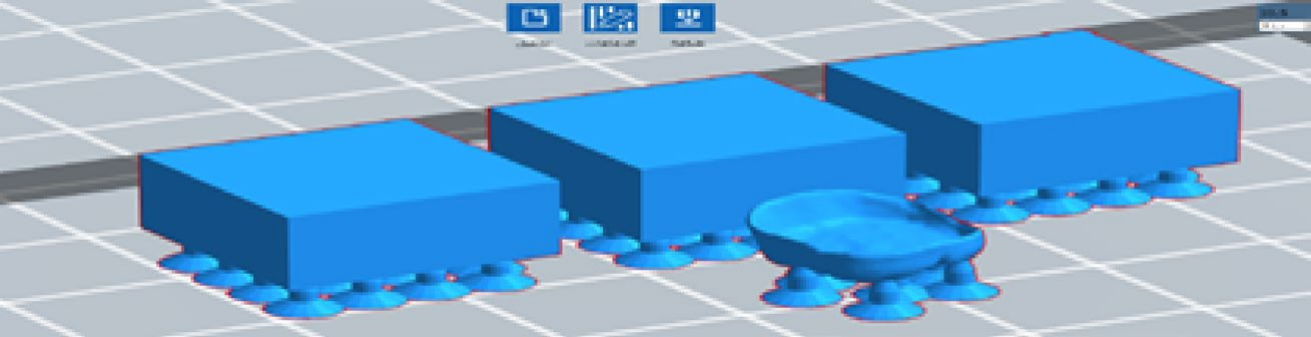
Samples starting point determination.

**Fig. 4:**
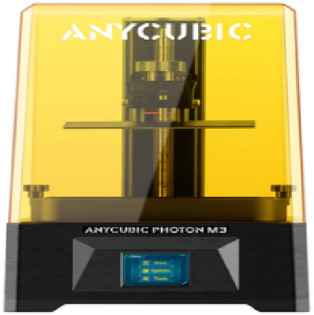
Photon M3 4 K + Any cubic, 55W 3d printer.

**Fig. 5:**
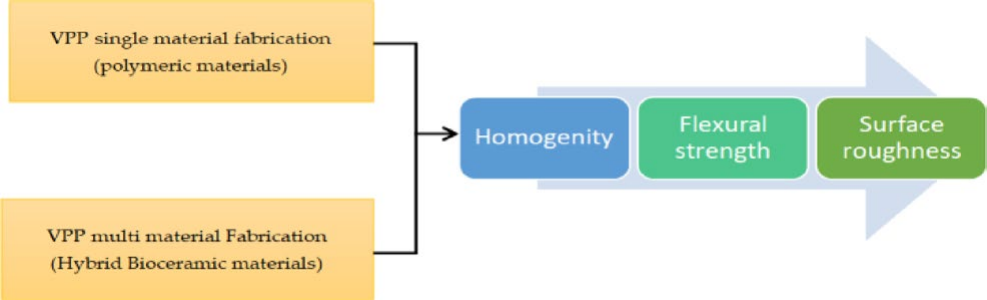
Printed part characterization methodology.

**Fig. 6:**
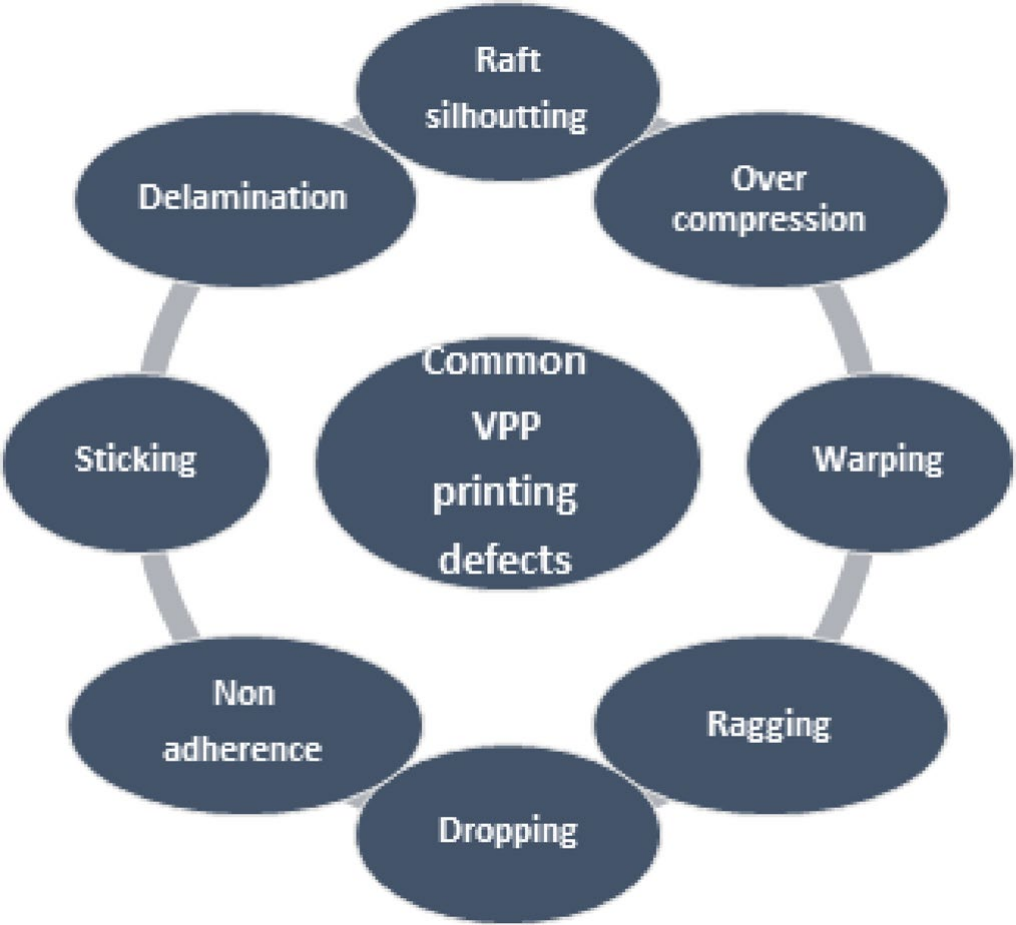
Common printing defects in the VPP process^30^.

The yttria content of the tetragonal phase can be calculated using the formula^22^

1 $${YO}_{1.5}\left(mol\%\right)= \frac{1.0225-\frac{c}{a\sqrt{2}}}{0.0016}$$

where a and c are the unit cell dimensions expressed in angstroms that are calculated in the case of the tetragonal phase by using the formula^23^

2 $$\frac{1}{{d}^{2}}=\frac{{h}^{2}+{k}^{2}}{{a}^{2}}+\frac{{l}^{2}}{{c}^{2}}$$

where d is the distance between planes in angstroms, and h, k, and l are the Miller indices; a and c are the unit cell dimensions.

The unit cell dimension of the tetragonal phase of our characterized powder is calculated by utilizing the data in Table 1 and Eq. 2. Then, we utilize the calculated unit cell dimension to calculate yttria content mol% of the tetragonal phase of our characterized powder by utilizing Eq. 1.

### Experimental design and setup

#### Data preparation

The stability of the ceramic colloid depends mainly on particle size, the viscosity of the colloid, and the ceramic powder gravity force according to simplified Navier–Stokes Eq. 3 in which terminal velocity (v_d_) minimization is an indicator of the ceramic colloid stability maximization^24^, and Where r is the radius and ρ is the density of the ceramic particles, ρ_0_ is the density of the resin, g is the acceleration, and ƞ is the viscosity of the liquid.

3 $${v}_{d}=\frac{2{r}^{2}(\rho -{\rho }_{^\circ })g}{9\eta }$$

Our study focuses on four types of ceramic colloids with ceramic powder weight percentages of 20 wt%, 10 wt%, 5 wt%, and 1 wt%, and based on Eq. 3 that is target is minimizing weight percentage of the ceramic powder to minimize its gravity force, and therefore maximizing its stability, then we uses Eq. 4 to calculate the ideal interparticle distance between nanoparticles (h) of the four proposed ceramic colloids which their data are shown in Tables 1, 2, 3 and 4. We then calculate the Griffith factor (Q) from Eq. (5), and then use the Griffith factor to calculate the Depth of penetration (layer thickness) of the laser power into ceramic colloid as it represents the capability of a matter to diffuse a radiation and it is an important parameter that should be determined before 3d printing to ensure the success of the printing. Where n_r_ is the refractive index of photosensitive resin, n_c_ is the refractive index of ceramic particles, D_p_ is the layer thickness, d_c_ is the particle size of ceramic particles, V_cm_ is the limited volume ratio that is equal to л/6, and λ wavelength of the irradiation, which is equal to 0.405 microns. We also calculate the normal exposure time from Eq. 7 and that time is the time required to solidify each layer.

**Table 2:** Data of three colloid types were obtained from Eqs. 3, 4, 5, and 3d printer technical data.

Colloid type	Colloid 1	Colloid2	Colloid3	Colloid4
Ceramic powder wt%	20	10	5.0	1.0
Stability	low	medium	high	Very high
Printing time	high	medium	low	Very low
Printed product quality	very low	low	medium	high
Strength	very high	high	medium	low

**Table 3:** The encoding data of the three colloid types were obtained from Table 1.

Colloid type	Colloid 1	Colloid2	Colloid3	Colloid4	Sum
Ceramic powder vol%	20.0	10.0	5.0	1.0	36
Stability	25	50	75	100	250
Printing time	100.0	75	50	25	250
Printed product quality	25	50	75	100	250
Strength	100.0	75	50	25	250

**Table 4:** Normalized data of the three colloid types that obtained from Table 2.

Colloid type	Colloid 1	Colloid2	Colloid3	Colloid4	Sum
Ceramic powder vol%	0.55	0.280	0.14	0.03	1
Stability	0.1	0.2	0.3	0.4	1
Printing time	0.4	0.3	0.2	0.1	1
Printed product quality	0.1	0.2	0.3	0.4	1
Strength	0.4	0.3	0.2	0.1	1

4 $$h= {d}_{c} \left(\sqrt[3]{\frac{{V}_{cm}}{{V}_{c}}}-1\right)$$

5 $$Q=\frac{h}{\lambda }{\left({n}_{c}-{n}_{r}\right)}^{2}$$

6 $${D}_{p}=\frac{2{d}_{c}}{3Q{V}_{C}}$$

7 $$t_{{exposure}} = \frac{{Required\;curing\;energy}}{{available\;power\;of\;the\;3d\;printer}}$$

#### Data optimization

EWM (Entropy Weight Method)^5^. In this method, m indicators and n samples are set in the evaluation, and the measured value of the ith samples for the jth indicators is recorded as yij, the first step is the standardization of measured values, the standardized value of the ith sample in jth indicator the is denoted as Pij, and its calculation method is as follows:

8 $$P_{{ij}} = \frac{{y_{{ij}} }}{{\sum\limits_{{\varvec{i} = 1}}^{\varvec{n}} {\varvec{y}_{{\varvec{ij}}} } }},$$

In the EWM, the entropy value Ej of the jth index is calculated as follows:

9 $$E_{j} = - \frac{{\sum\limits_{{\varvec{i} = 1}}^{\varvec{n}} {\sum \left( {\varvec{p}_{{\varvec{ij~~}}} \varvec{*ln}\left( {\varvec{p}_{{\varvec{ij}}} } \right)} \right)} }}{{\varvec{ln}\left( \varvec{n} \right)}},$$

In the EWM, the calculation method of weight wj is as follows:

10 $$W_{j} = \frac{{1 - E_{j} }}{{\sum\limits_{{\varvec{J} = 1}}^{\varvec{m}} {\left( {1 - \varvec{E}_{\varvec{j}} } \right)} }},$$

The weights calculated from EWM are used in the FUZZY-TOPSIS method to get the most suitable materials, and its algorithm is sequenced as follows:

**Step 1:** Create a decision or evaluation matrix ***D***.

The matrix consists of n samples (A1,…, Am) and m criteria (y1,…, ym), with its element y_ij_ , where i =  (1,…………., n) and j = (1,……………., m):

11 $$D= {\left[\begin{array}{ccc}{y}_{11}& \cdots & {y}_{1m}\\ \vdots & \vdots & \vdots \\ {y}_{n1}& \cdots & {y}_{nm}\end{array}\right]}_{nXm},$$

**Step 2:** Construct the normalized decision matrix ***R***:

12 $$r_{{ij}} = \frac{{y_{{ij}} }}{{\sqrt {\sum\limits_{{\varvec{i} = 1}}^{\varvec{n}} {\varvec{y}_{{\varvec{ij}}} ^{2} } } }},$$

13 $$R= {\left[\begin{array}{ccc}{r}_{11}& \cdots & {r}_{1m}\\ \vdots & \vdots & \vdots \\ {r}_{n1}& \cdots & {r}_{nm}\end{array}\right]}_{nXm},$$

**Step 3:** Construct the weighted normalized decision matrix ***V***.:

14 $$\left[\text{V}\right]=\left[{W}_{j}{r}_{ij}\right],$$

A set of weights W = (${w}_{1}$,……….,${w}_{m}$) and $\sum_{j=1}^{m}{w}_{j }=1,where\;{w}_{j}>0,$

$j = 1, \ldots \ldots ,m\;is\;given\;to\;the\;coeereponing\;criterion\;y_{j}$, Where j = 1,……..,m. The matrix V = [*w*_j_r_*ij*_] is calculated by multiplying the elements as each column of the matrix R by their associated weights *w*_*j*_, * j* = 1,.......,m

15 $$V= {\left[\begin{array}{ccc}{v}_{11}& \cdots & {v}_{1m}\\ \vdots & \vdots & \vdots \\ {v}_{n1}& \cdots & {v}_{nm}\end{array}\right]}_{nXm},$$

**Step 4:** determination of the positive ideal and negative –ideal solution ${V}^{+}$(PIS) and ${V}^{-}$(NIS), respectively.

PIS is defined as:

16 $$V^{ + } = \left\{ {v_{1}^{ + } , \ldots \ldots ,v_{m}^{ + } } \right\} = \left\{ {\left( {\begin{array}{*{20}c} {\max } \\ i \\ \end{array} \;v_{ij} |j{ } \in J} \right),\left( {\left( {\begin{array}{*{20}c} {\min } \\ i \\ \end{array} \;v_{ij} |j{ } \in J^{\prime}} \right)} \right)} \right\},$$

And NIS is defined as:

17 $${V}^{-}=\left\{{{v}_{1}}^{-},\dots \dots ,{{v}_{m}}^{-}\right\}=\left\{\left(\begin{array}{c}\text{min}\\ i\end{array} {v}_{ij}|j \in J\right),\left(\left(\begin{array}{c}\text{max}\\ i\end{array} {v}_{ij}|j \in {J}^{\prime}\right)\right)\right\},$$

where j is associated with the benefit criteria and ${j}^{\prime}$ is associated with the cost criteria, on criterion ${y}_{j},where\;j=1,\dots \dots .,m\;for\;all\;samples\;i=1,\dots \dots ..,n.$

**Step 5:** Calculate the separation measure between alternative ${A}_{i}$ (samples), $s_{i} ^{ + }$ $and$ $s_{i} ^{ - } ,$ $and$ $the$ $ideal$ $and$ $the$ $negativ$ $ideal$ $solutions,$ $respectively.$

The separation measure or distance between alternatives (samples) and the PIS can be measured by n-dimensional Euclidean distance as follows

18 $$S_{i} ^{ + } = \sqrt {\sum\limits_{{\varvec{j} = 1}}^{\varvec{m}} {\left( {\varvec{V}_{{\varvec{ij}}} - \varvec{V}_{\varvec{J}} ^{ + } } \right)^{2} } } ,$$

For alternatives $${A}_{i} ,$$ i = 1,…….,n

The separation measure or distance between alternatives (samples) and the NIS can be illustrated as follows:

19 $$S_{i} ^{ - } = \sqrt {\sum\limits_{{\varvec{j} = 1}}^{\varvec{m}} {\left( {\varvec{V}_{{\varvec{ij}}} - \varvec{V}_{\varvec{J}} ^{ - } } \right)^{2} } } ,$$

For alternatives $${A}_{i}$$, i = 1,……., n

**Step 6**: Calculate the relative closeness ${{C}_{i}}^{*}$ of alternatives ${A}_{i},$ i = 1,…….,n, the relative closeness or ranking index of samples is defined as follows:

20 $${{\mathbf{C}}_{\mathbf{i}}}^{\mathbf{*}}=\frac{{{\mathbf{S}}_{\mathbf{i}}}^{-}}{{{\mathbf{S}}_{\mathbf{i}}}^{+}+{{\mathbf{S}}_{\mathbf{i}}}^{-}}$$

The larger the index value is, the better is the performance of the alternative. The relative closeness is the judgment rule of the decision inFUZZY-TOPSIS.

**Step 7:** Rank the preference order of all alternatives.

A set of alternatives ${A}_{i}$, *i* = 1,…, *n* can now be preference ranked according to the descending order of the value of ${{c}_{i}}^{*}$. In general, the selection should be the alternative with the highest value of the relative closeness, but it is claimed that if the relative closeness of the alternative is less than 0.5, it is rejected and previous steps are repeated until the trial in which all alternative is greater than or approaches 0.5, then at that trial data is prepared and ready for machine learning model.

### Indirect mixing strategy

The target of the indirect mixing process is stabilizing the ceramic colloid that its components are shown in Fig. 2. The stabilization process is a function of three steps that the adsorption process in which the effective area of the particulate surface must be adequately covered by dispersant molecules: semblance or displaying similarity to the liquid phase by a portion of the dispersant molecule directed outwards into that phase; and isolation in which a barrier must be created around each particle to prevent other particles from coming into direct contact^25^. So we mix three constitutions indirectly in a homogenizer (dihane HG—15D, South Korea) that are yttria-stabilized zirconia as c1, disperbyk111 as c2, and commercial polymeric resin as c3, we mix 1wt% c1 with 1wt% c2 for 15 min to form mixture 1, then we mix 97wt% c3 with 1wt% c2 for 6 min to form mixture 2, after that we mix mixture 1 with mixture 2 for 21 min to form mixture 3, After that, sonication of the mixture 3 for 3 min that is the final ceramic colloid, we use zeta potential that is the basic surface charge divided by the distance between the surface and the random solution-phase ionic atmosphere as a mean to measure and characterize the stability of the colloid based on data shown in Table 5.

**Table 5:** Stability behavior of the ceramic colloid according to zeta potential values^33^.

Zeta potential (mv)	Stability behavior of the ceramic colloids
From 0 ± to 5	Rabid coagulation or flocculation
From 10 ± to 30	Incipient stability
From 30 ± to 40	Moderate stability
From 40 ± to 60	Good stability
More than ± 61	Excellent stability

### Additive manufacturing of a concurrent bioceramic hybrid colloid

The first step in 3d printing process is designing the CAD file and converting it into STL file using inventor software for the uniform samples, for samples with complex geometry we used 3d scanner to generate STL file. After that, we used flash DLP print software to put start point of the samples shown in Fig. 3 to be ready for bottom to up 3d printing, then we using Foreware software to feed STL files of the samples into 3d printer shown in Fig. 4. After that layer thickness is adjusted to 25 micron as it represents the distance that the platform will move from upper to lower^26^, then a ultraviolet (UV) with 405nm is used to cure the resin layer by layer until part is completed with bottom exposure time 80 s and normal exposure time greater than 10 s for each layer as 10 s for each layer was too short to solidify the bioceramic colloid^27,28^, after that the green part is characterized according to Fig. 5 taking into consideration that all printing defects shown in Fig. 6 should be avoided.

### Bioceramic dental crowns cost estimation

**Fig. 7:**
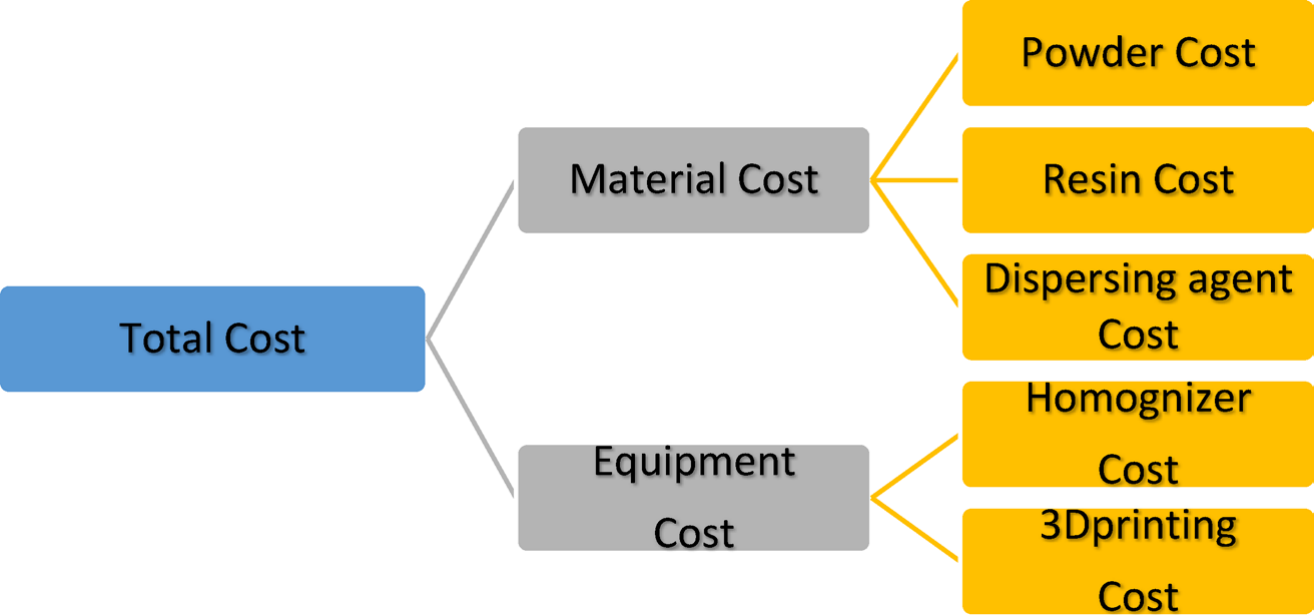
Total manufacturing cost.

Bioceramic dental crowns is a function of the following parameters: powder cost in $ per gram, number of powder grams per piece, resin cost in $ per gram, number of resin grams per piece, dispersing agent cost in $ per gram, number of dispersing agent grams per piece, weight of the piece, homogenizer cost per minute, homogenizer time inminutes, 3d printing cost per gram. We use the previous parameters to estimate the cost of bioceramic dental crown, and the cost Parameters a viewed in Table 6, and the manufacturing cost is estimated based on Eqs. 21, 22, 23, and its approach is shown in Fig. 7.

**Table6:** Bio ceramic additive manufacturing cost parameters.

Material Cost in $		Equipment Cost in $		Weight of the piece in grams		Total Cost in $	
Powder cost in $ per gram	P_c_	3d printing cost per gram	PC_c_	Weight percentage of powder	W_p_	Material Cost	Cm
Number of powder grams per piece	N_p_						
Resin cost in $ per gram	R_c_	Homogenizer cost per minute	H_c_	Weight percentage of resin	W_r_	Equipment Cost	Ce
Number of resin grams per piece	N_r_						
Dispersing agent cost in $ per gram	D_c_	Homogenizer time in minutes	t_h_	Weight percentage of dispersing agent	W_d_	Total Cost	CT
Number of dispersing agent grams per piece	N_d_			Total weight	TW

21 $${\varvec{C}}{\varvec{T}}={{\varvec{C}}}_{{\varvec{m}}}+{{\varvec{C}}}_{{\varvec{e}}}$$

22 $${{\varvec{C}}}_{{\varvec{m}}}={{\varvec{P}}}_{{\varvec{c}}}\boldsymbol{*}{{\varvec{N}}}_{{\varvec{p}}}+{{\varvec{R}}}_{{\varvec{c}}}\boldsymbol{*}{{\varvec{N}}}_{{\varvec{r}}}+{{\varvec{D}}}_{{\varvec{c}}}\boldsymbol{*}{{\varvec{N}}}_{{\varvec{d}}}$$

23 $${{\varvec{C}}}_{{\varvec{e}}}={{\varvec{H}}}_{{\varvec{c}}}\boldsymbol{*}{{\varvec{t}}}_{{\varvec{h}}}+{{\varvec{P}}{\varvec{C}}}_{{\varvec{c}}}\boldsymbol{*}{\varvec{T}}{\varvec{W}}$$

## Results and discussion

### Ceramic powder characterization

**Fig. 8:**
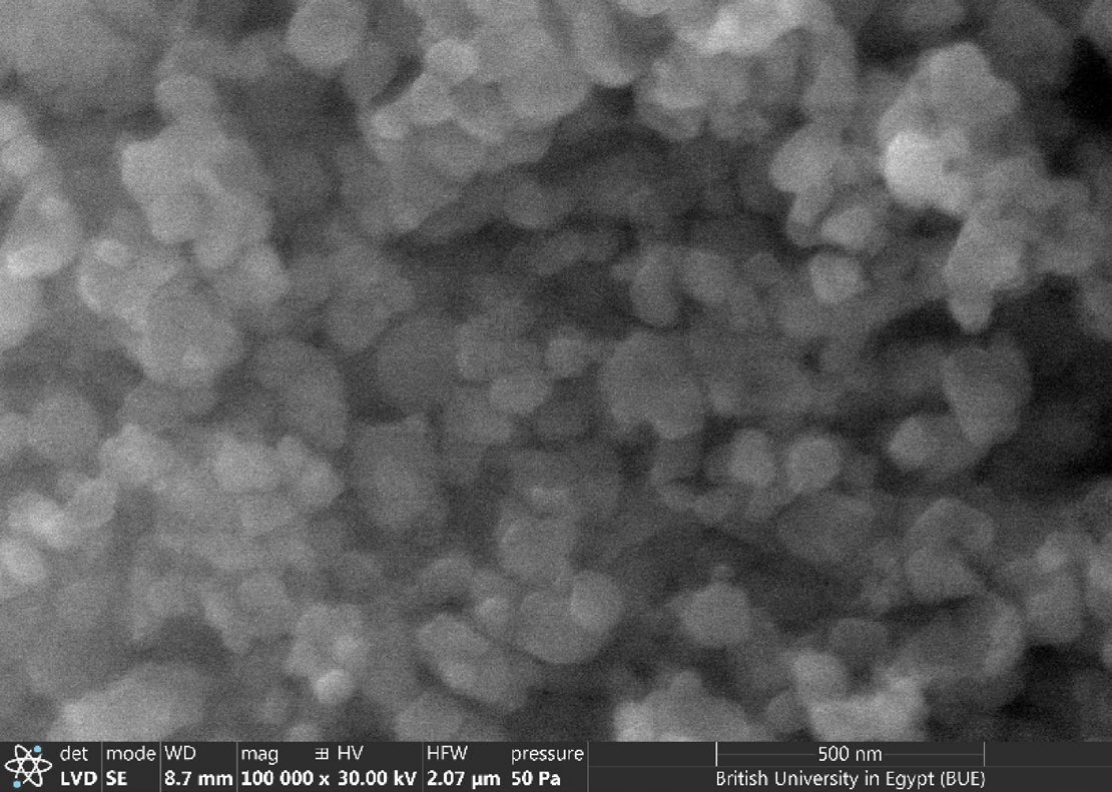
FESEM analysis of zirconia powder.

**Fig. 9:**
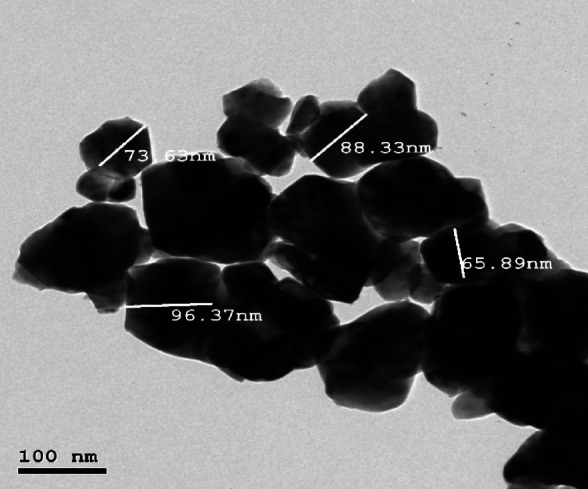
HRTEM analysis of zirconia powder.

**Fig. 10:**
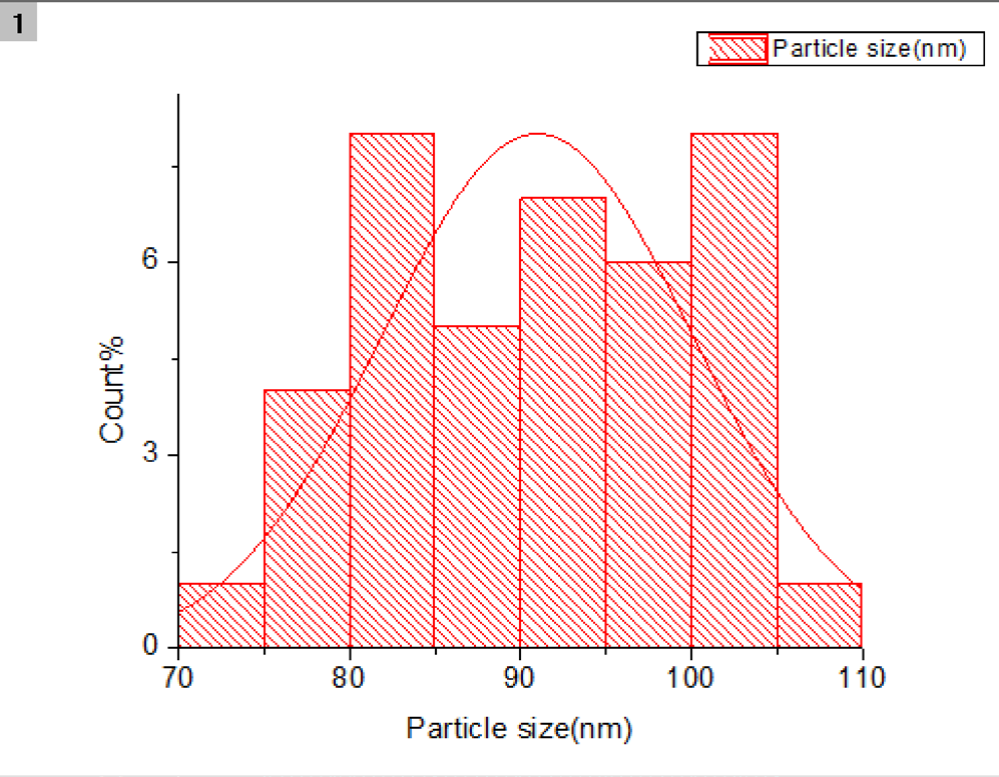
FESEM normally particle size. Distribution of zirconia powder.

**Fig. 11:**
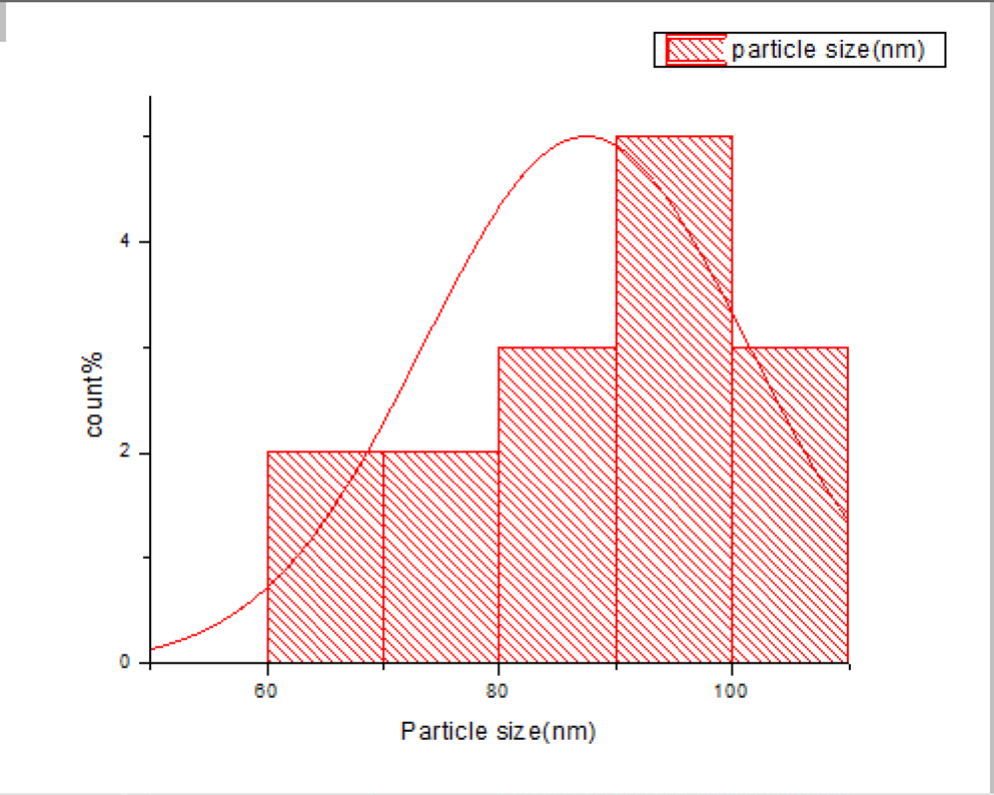
HRTEM Normally particle size. Distribution of zirconia powder.

Figures 8, 9 show the particle size of a variety of zirconia powder particles, and data in them are extracted and statistically analyzed using Imagej and origin pro softwares and their results are shown in Figs. 10 and 11 in which the particle size is indicated with an average of 91nm (Table 7).

**Table 7:** Analysis of the peaks of Figs. 12, 13, and phase identification*.*

Peak number	Position _°2θ_	d-spacing _Å_	FWHM Total _°2θ_	Miller indices (h k l)	Crystal system
1	24.31	3.66	0.65	011	monoclinic
2	28.28	3.15	0.33	− 111	monoclinic
3	30.28	2.95	0.26	101	tetragonal
4	31.49	2.84	0.40	111	monoclinic
5	34.77	2.58	0.38	002	tetragonal
6	35.25	2.54	0.31	110	tetragonal
7	40.82	2.21	0.19	− 112	monoclinic
8	43.08	2.10	0.31	102	tetragonal
9	50.39	1.81	0.57	− 221	monoclinic
10	55.56	1.65	0.57	− 311	monoclinic
11	59.48	1.55	0.45	103	tetragonal
12	60.11	1.54	0.40	− 203	monoclinic
13	62.81	1.48	0.42	202	tetragonal
14	65.45	1.42	0.66	023	monoclinic
15	73.18	1.29	0.40	004	tetragonal
16	74.38	1.27	0.37	220	tetragonal
17	81.91	1.18	0.88	213	tetragonal
18	83.86	1.15	0.89	114	tetragonal
19	84.90	1.14	0.55	222	tetragonal

**Fig. 12:**
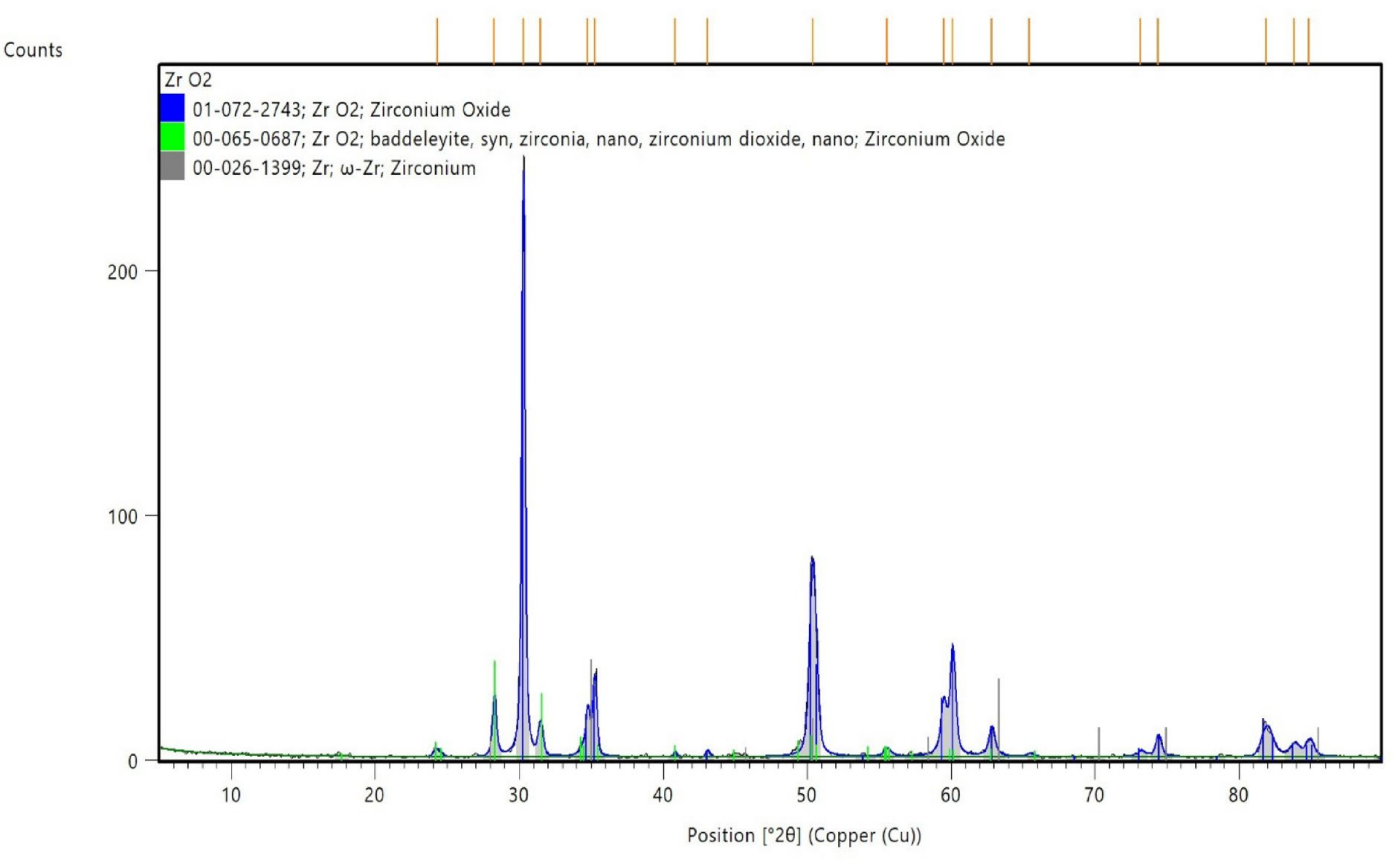
XRD analysis of zirconia powder.

**Fig. 13:**
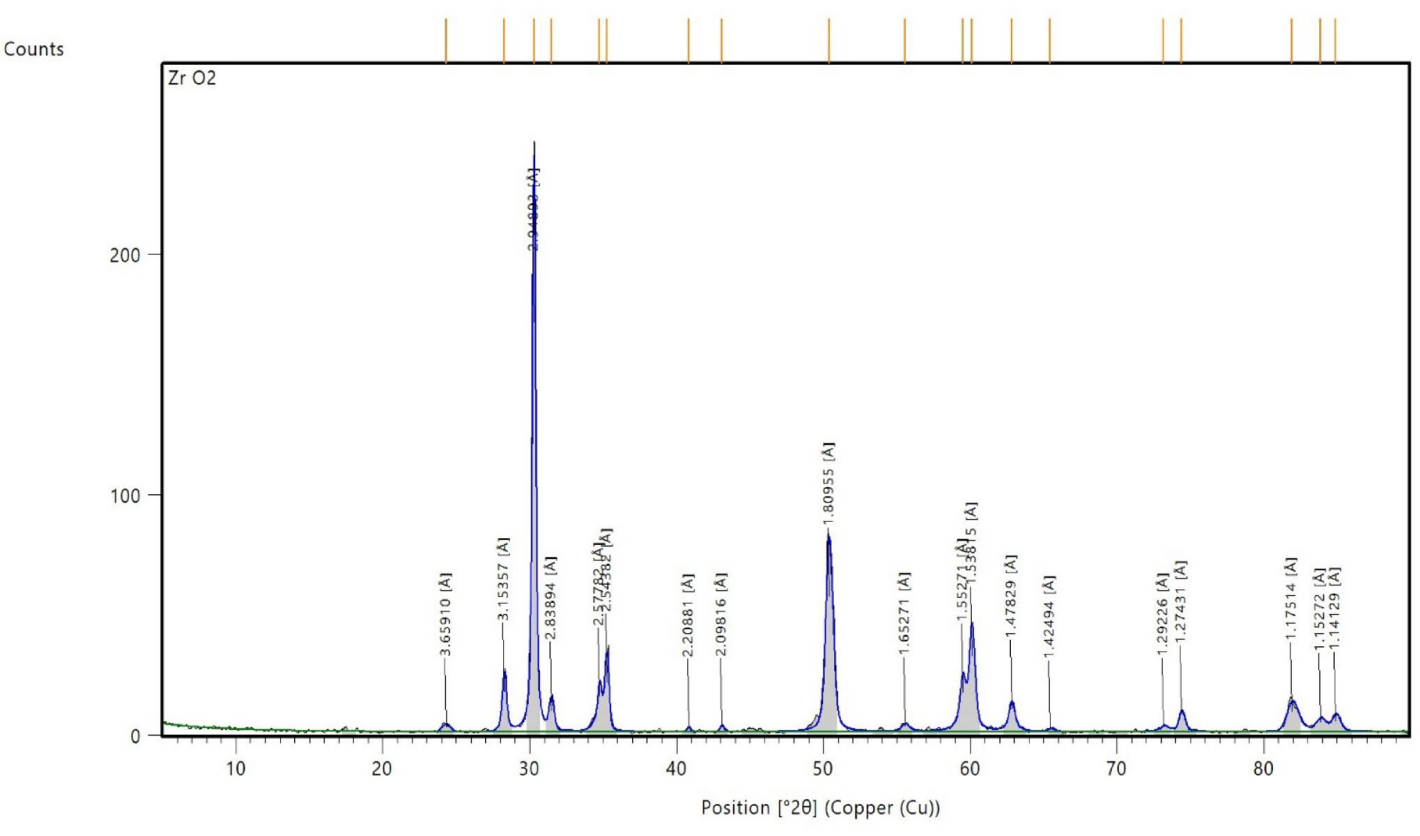
XRD analysis of zirconia powder.

To calculate the yttria content of the utilized powder we extract data from Figs. 12, 13 to form Table 8, then utilizing data in Table 7 and Eq. (2) to calculate the unit cell dimensions then using Eq. (1) to calculate the yttria content in ceramic powder that is 4 mol%.

**Table 8:** Unit cell dimensions in Angstrom of the utilized powder in tetragonal phase region*.*

Unit cell (Angstrom)	Value(Ǻ)
a	3.59
c	5.16

### Ceramic colloid performance parameters optimization

Table 9 indicates the weights of ceramic colloid performance parameters that are calculated using fuzzy-entropy method, and we noticed that the larger weights are with layer thickness and ceramic powder volume percentage. Table 10 indicated the criteria indices of the three proposed ceramic colloids, and it is noticed that the ceramic colloid with 25 micron layer thickness and 1 ceramic powder wt% is the best choice.

**Table 9:** Weights of ceramic colloid performance parameters calculated by fuzzy-entropy method[34, 35].

Performance parameter	ceramic powder vol%	stability	printing time	printing quality	strength
Weight	0.4368	0.1408	0.1408	0.1408	0.1408

**Table10:** Criteria indices of the three proposed ceramic colloids.

Ceramic colloid	Colloid 1	Colloid2	Colloid3	Colloid4
Ceramic powder wt%	20	10	5	1
C*	0.2239	0.5219	0.7272	0.7761

### Indirect mixing stability measurement

**Fig. 14:**
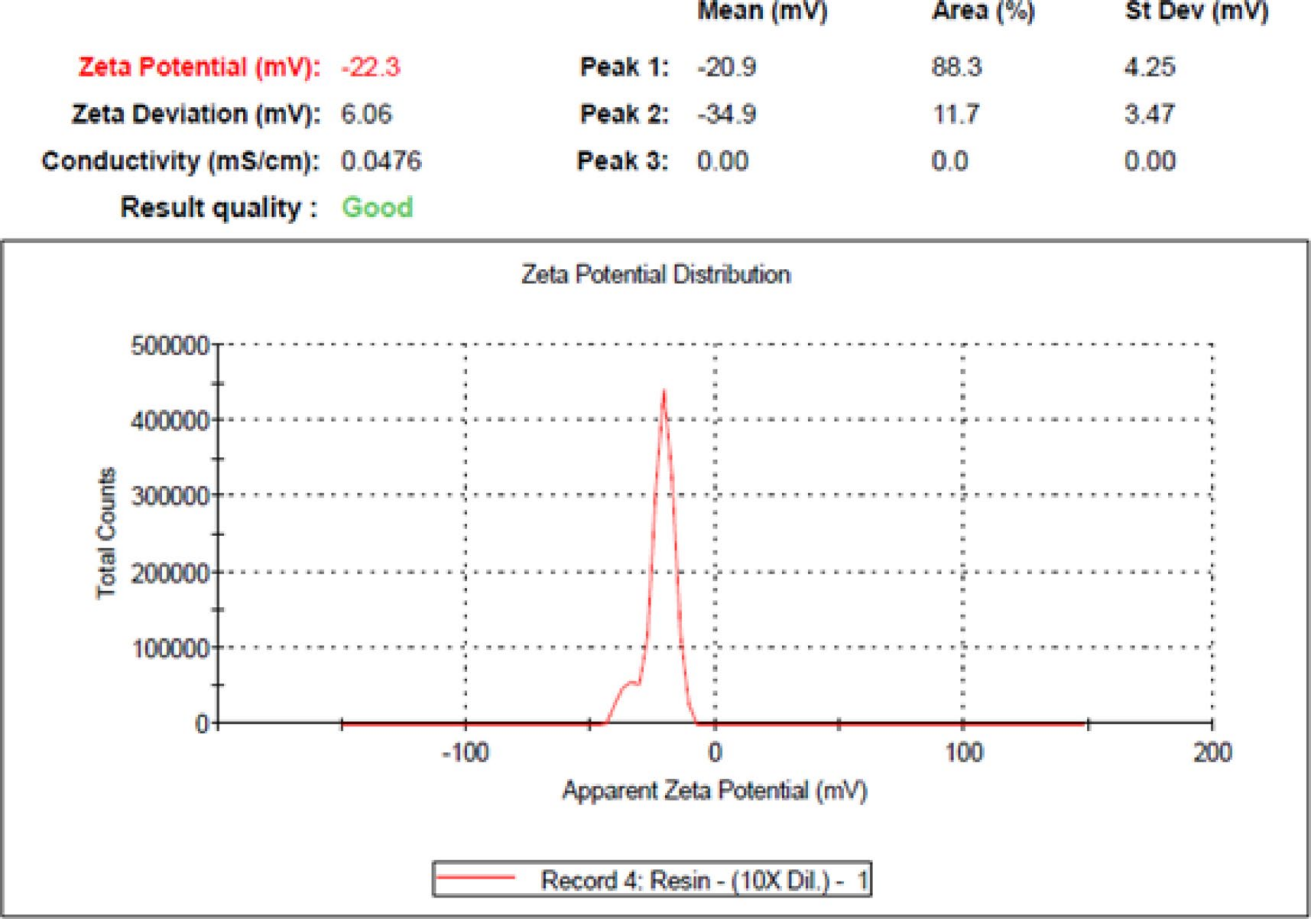
Indication of zeta potential measurement of the sample with the incipient stability.

**Fig. 15:**
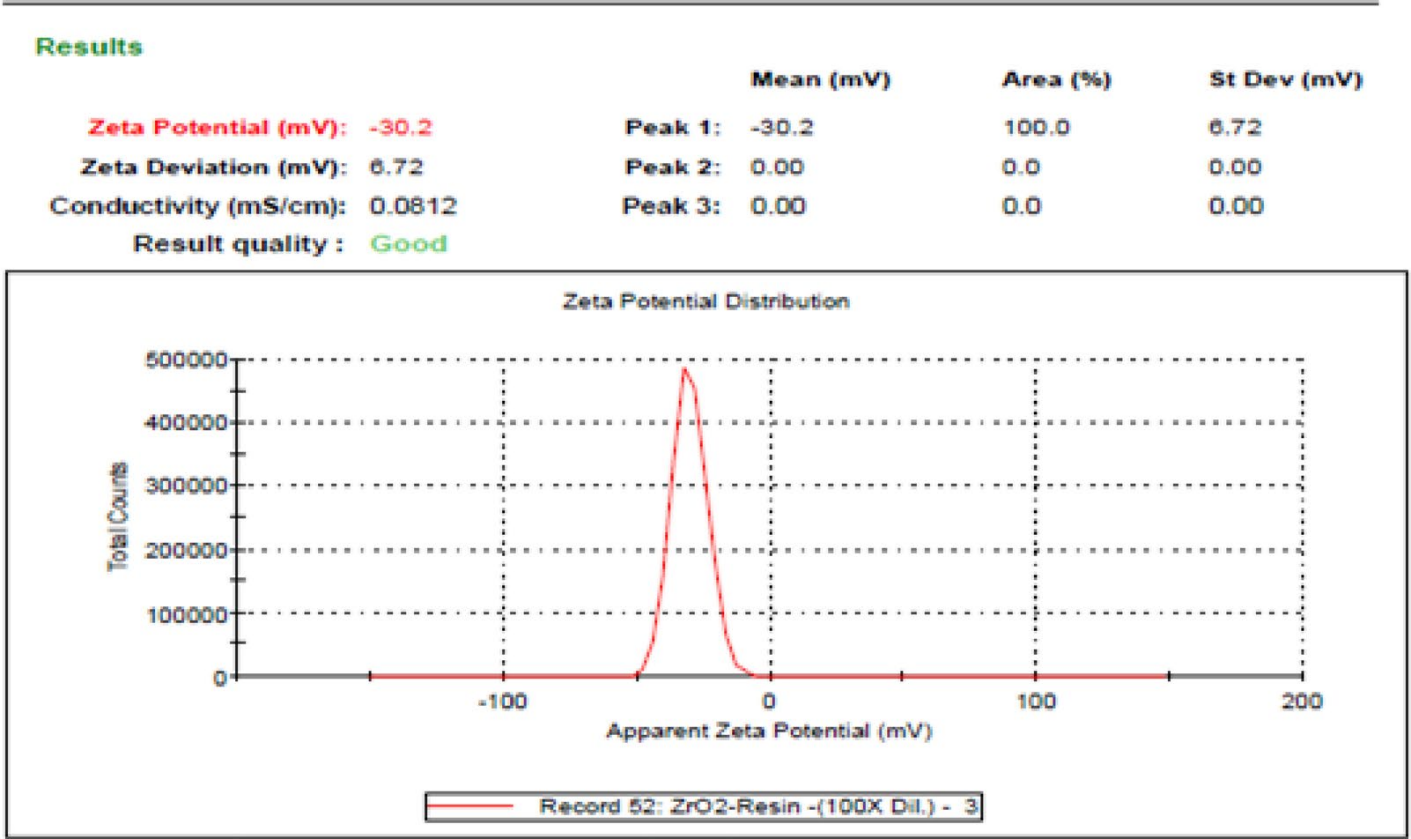
Indication of zeta potential measurement of the sample with the moderate stability.

After indirect mixing the optimum ceramic colloid, we measured its zeta potential, and the results are indicated in Fig. 14. We noticed that the behaviour of ceramic colloid with 10 wt% ceramic powder and 7 wt% dispersing agent is in the incipient stability region., and we also noticed that the behaviour of ceramic colloid with 1 wt% ceramic powder and 2 wt% dispersing agent is in the moderate stability region that is shown in Fig. 15, and this improvement in the stability behaviour is due to two factors that are optimizing the dispersing agent weight percetage that is advisible to be greater than 1 wt% and less than 7 wt%, and the other factor is minimizing the ceramic powder percentage that also leads to improve the stability of the ceramic colloid.

### 3D printing of the colloid

**Fig. 16:**
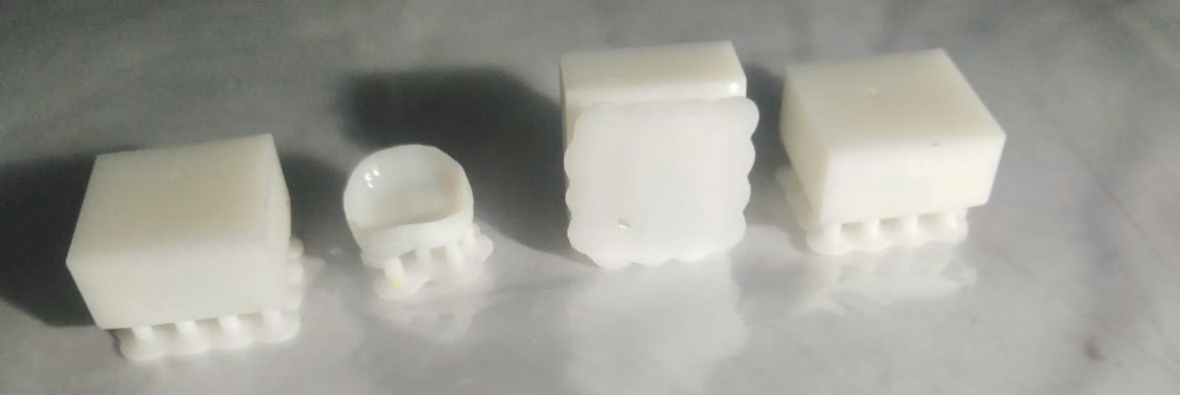
Printed parts from polymeric commercial resin.

**Fig. 17:**
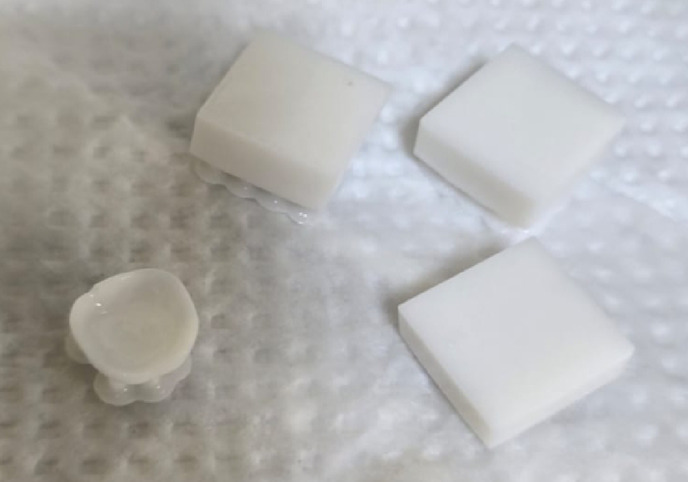
Printed parts from hybrid ceramic resin.

**Fig. 18:**
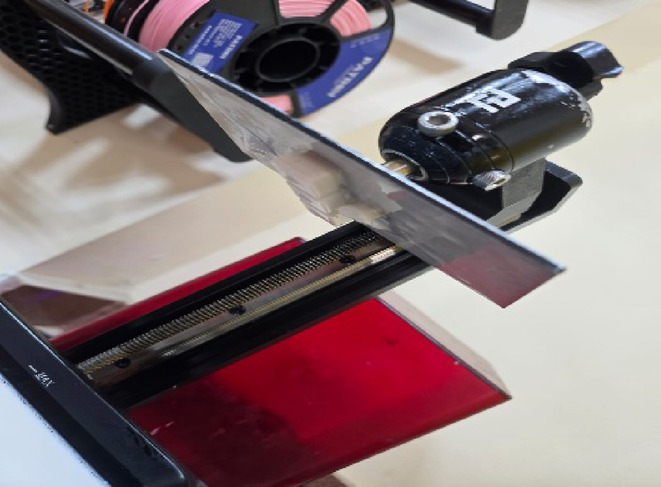
Hybrid ceramic printed parts on the print plate.

**Fig. 19:**
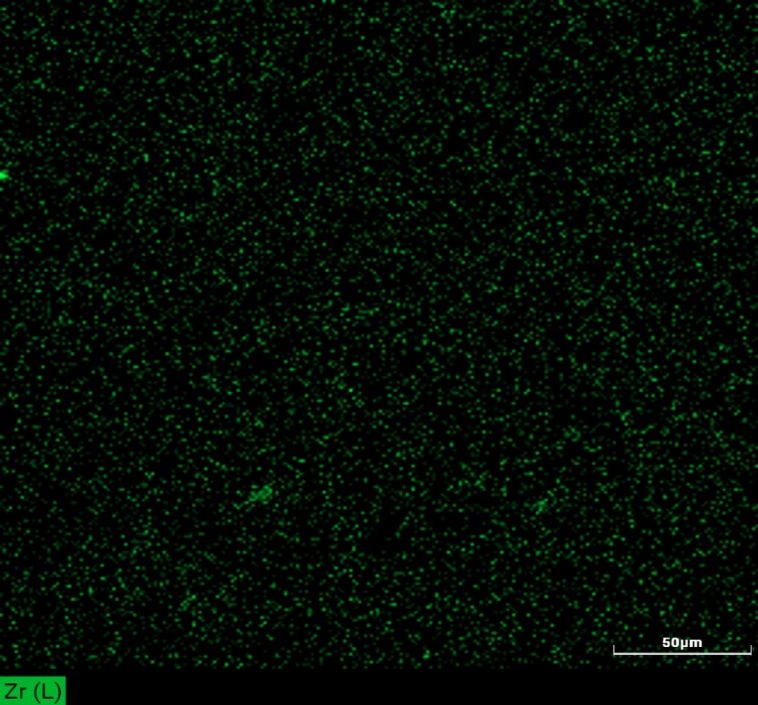
Zirconia distribution in the 3d printed part using SEM.

**Fig. 20:**
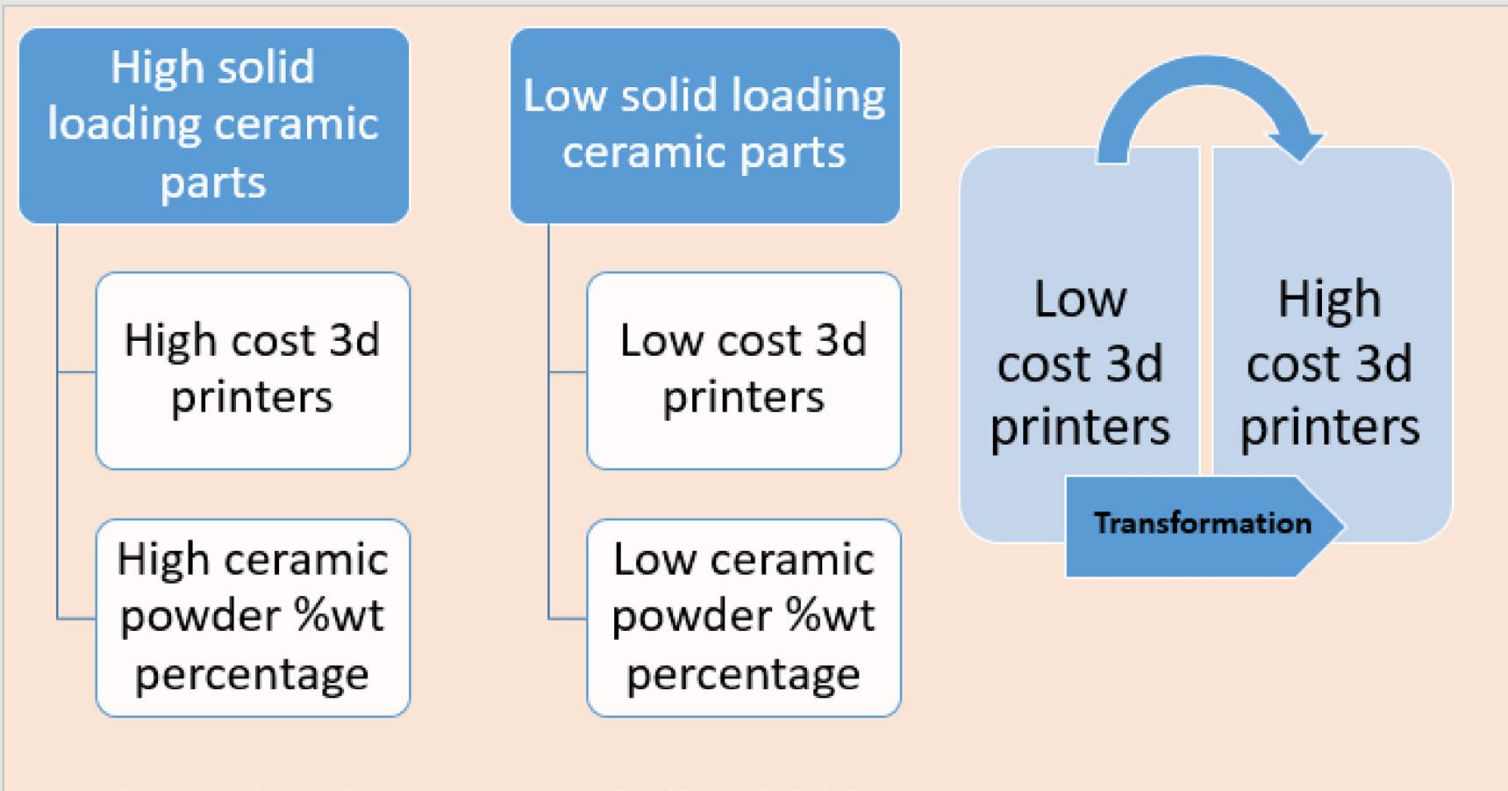
The challenge of transforming low-cost 3d printers into high-cost 3d printers.

The printed parts of the polymeric resin using photon M3-4 K 3d printer with 50 micron layer thickness and 4 s exposure time are shown in Fig. 16^29^, and Figs. 17, 18 show that the hybrid ceramic printed parts using elegoo mars 2pro 3d printer with 15 s exposure time 15 s as it is the time required for curing ceramic colloid without printing defects, 25 micron layer thickness, 80 s bottom exposure time, 8 bottom layers count, retract speed 60 mm/min, bottom lift speed 15 mm/min, lifting speed 30 mm/min as the previous printing parameters are the optimum parameters required for printing, taking into consideration that decreasing exposure time to less than 15 s and increasing retract speed over than 60 mm/min, increasing bottom lift speed over than 15 mm/min, and increasing lifting speed over than 30 mm/min lead to possibility of the printing defects such as incomplete curing in which printing process fails due to insufficient energy required for curing, printed parts don’t stick to print plate due in sufficient bottom exposure time, and excessive printing speeds (Figs. 19, 20).

### Characterization of the 3D printed part

The characterization of the 3d printed part indicated that the homogenity of the product is very high and the zirconia powder is well dispersed and that exactly affects postively on mechanical performace of the printed products as the ceramic powder repersents the fiber of the composite materials that improves the mechanical performance of the printed products(Fig. 19). 

### Bioceramic dental crowns cost estimation

Bioceramic dental crowns traditionally manufactured by subtractive technology such as CNC milling and the bioceramic dental crown manufacturnig cost of this method is high that is nearly $20 as well as existing of powder waste, on the other hand bioceramic additive manufacturing is a good alternative to subtractive methods due to lower cost and waste minimization and in the following Tables 11, 12 we show the bioceramic additive manufacturing cost estimation of the dental crown. All the data cost is gathered experimentally and actually from the egyptian market, taking into considerantion that the actual cost of polymeric dental crown additively manufactired is $5 in dental market in Egypt, and the cost of the bioceramic dental crowns manufactured by our approach is $ 2.04 that is lower than polymeric dental crowns existing in egyptian market with better mechanical properties than it(Figs.  20).

**Table11:** Bioceramic Additive Manufacturing cost estimation.

Material Cost in $		Equipment Cost in $		Weight of the piece in grams		Total Cost in $	
P_c_	0.76	PC_c_	0.1	W_p_	0.025	Cm	0.0875
N_p_	0.025						
R_c_	0.02	H_c_	0.04	W_r_	2.425	Ce	1.93
N_r_	2.425						
D_c_	0.4	t_h_	42	W_d_	0.05	CT	2.0175
N_d_	0.05			TW	2.5

**Table12:** Concurrent Bioceramic Additive Manufacturing cost estimation.

Material Cost in $		Equipment Cost in $		Weight of the piece in grams		Total Cost in $	
P_c_	0	PC_c_	0.1	W_p_	0.025	Cm	0.0685
N_p_	0.025						
R_c_	0.02	H_c_	0.04	W_r_	2.425	Ce	1.93
N_r_	2.425						
D_c_	0.4	t_h_	42	W_d_	0.05	CT	1.9985
N_d_	0.05			TW	2.5		

## Conclusion

This study aimed to present a concurrent engineering approach that utilizes ceramic powder waste and reuses it for bioceramic additive manufacturing. The novelty of our approach started with ceramic powder characterization to determine its mean particle size, crystalline structure, and unit cell dimensions; then Bioceramic colloid design in which we design a ceramic colloid based on optimization of the most significant dependent parameters that affect the performance of the colloid that are stability of the colloid and capability of the 3d printer, and these dependent parameters are a function of some independent parameters that are ceramic powder volume percentage, layer thickness, particle size, scattering efficiency, refractive index of the ceramic powder, refractive index of the resin, viscosity of the colloid, dispersing agent weight percentage, and mixing strategy. After that, we use an indirect mixing strategy to get the required bioceramic colloid that is fed into a 3d printer to get the additively manufactured parts, then we test the homogeneity of the printed product to check its quality. This research is valuable to all manufacturers working on ceramic additive manufacturing as a method to produce high-quality 3d printed products with the application of concurrent or sustainable engineering. So we will state concisely our findings with limitations of our study and suggestions for future research in the following points:-

1. Utilization of the ceramic powder waste extracted from subtractive manufacturing reduces the cost of the basic raw material, that is, the ceramic powder, and also saves the ball milling cost that manufacturers use to reduce the particle size of the ceramic powder.
2. Ceramic powder characterization is a critical step that is needed to determine the particle size of the ceramic powder that whose mean is 91 nm in our study, and it is an independent parameter that controls the ceramic colloid stability, and the required layer thickness for additive manufacturing.
3. Ceramic powder characterization is also a critical step that is needed to determine the crystalline structure of the ceramic powder to determine the phases of the ceramic powder, and in our study the tetragonal phase is the common phase, and the unit cell dimensions are calculated to determine the mole percentage of yttria in the ceramic powder that is equal to 4 mol% in our study.
4. The stability of the ceramic colloid does not depend only on ceramic powder vol% and dispersing agent wt% but other parameters should be taken into consideration, such as gravitational force difference between the resin as a solvent and the ceramic powder as a solute, mixing strategy, viscosity of the colloid, and particle size.
5. Achieving good stability occurs when minimizing the gravitational force of the ceramic powder.
6. The stability of the ceramic colloid is a good indicator of additive manufacturing success or failure.
7. 3d-Printing success depend mainly on adjusting a suitable layer thickness with suitable ceramic powder vol% based on Eq. (4), also adjusting exposure time shown in Eq. (7) greater than 10 s as it is too short to solidify the initial bioceramic colloid, slowing down the print speed for the first layer to 50% of regular print speed to avoid the problem of dropping, checking the expired date of the resin to check its availability to avoid ragging problem.
8. The homogeneity of the additively manufactured product is a good indicator of the product quality.
9. Our work lacks mechanical performance clarification and clinical views that would be postponed to future research.
10. The use of the commercial 3d printer that is used for polymer resin additive manufacturing in ceramic additive manufacturing is a limitation in our study as its printing capability is limited and it prints only ceramic colloid with low powder volume percentage but this limitation could be treated by some modifications in the design of the 3d printer that should be taken into consideration in the future research as shown in Fig. 17
11. The estimated cost of the concurrently additively manufactured dental crowns is much lower than that of subtractive manufactured ceramic dental crowns and additively manufactured polymeric dental crowns.

## Data Availability

The data presented in this study are available on request from the corresponding author.
